# Rapid Spread of Pneumococcal Nonvaccine Serotype 7C Previously Associated with Vaccine Serotype 19F, England and Wales

**DOI:** 10.3201/eid2410.180114

**Published:** 2018-10

**Authors:** Ashley Makwana, Shamez N. Ladhani, Georgia Kapatai, Ella Campion, Norman K. Fry, Carmen Sheppard

**Affiliations:** Public Health England’s National Infection Service, London, UK (A. Makwana, S.N. Ladhani, G. Kapatai, E. Campion, N.K. Fry, C. Sheppard);; St. George’s University of London, London (S.N. Ladhani)

**Keywords:** invasive pneumococcal disease, pneumococcal conjugate vaccines, serotype, *Streptococcus pneumoniae*, replacement disease, capsular switching, serotype 7C, sequence type, ST177, England, Wales, United Kingdom, bacteria

## Abstract

We observed a sudden and rapid increase in rare invasive pneumococcal disease serotype 7C, from an annual average of 3 cases during 2000–01 through 2015–16 to 29 cases in 2016–17. The increase was caused almost entirely by clonal expansion of sequence type 177, previously associated with vaccine serotype 19F.

The bacterium *Streptococcus pneumoniae* is a major global cause of meningitis, septicemia, and pneumonia and is associated with high rates of illness and death. In September 2006, the childhood immunization program in the United Kingdom introduced PCV7, a pneumococcal conjugate vaccine against the 7 most common serotypes causing invasive pneumococcal disease (IPD) in children; a 13-valent PCV replaced it in April 2010 (*1*). Both vaccines have been associated with rapid and widespread declines in IPD across all age groups and, despite an increase in other disease from non-PCV serotypes, overall IPD rates have dropped by 56% and have remained lower than prevaccine rates (*1*).

In England and Wales, Public Health England (PHE) conducts enhanced national surveillance for IPD (*1*). National Health Service laboratories routinely report clinically significant infections to PHE and submit invasive isolates to the PHE National Reference Laboratory for serotyping (*2*). We observed an unusual increase in invasive isolates serotyped as 7C early in 2017 that continued into the 2017–18 epidemiologic year. We analyzed the epidemiology, clinical characteristics, genetic epidemiology, and outcomes of serotype 7C IPD since 2000–01 in England and Wales.

## The Study

We performed whole-genome sequencing (WGS) on 44 of 66 invasive 7C isolates collected during July 2005–June 2017, as well as 37 of 42 isolates collected during July 2017–January 31, 2018. We used previously published methods for WGS analysis (*3*,*4*). We used Bowtie version 2 (*5*) to map WGS reads to sequences for 1,689 antimicrobial-resistant genes in the ARG-ANNOT database (*6*). We used PBP typing to predict β-lactam resistance (*7*). Using MEGA7 (*8*), we drew a neighbor-joining tree (*9*) based on single-nucleotide polymorphism (SNP) alignments, as previously described (*10*). We included PHE data available on the European Nucleotide Archive (https://www.ebi.ac.uk/ena) (Technical Appendix Table) and used a non–sequence type (ST) 177 serotype 19F (Taiwan 19F-14; GenBank accession no. NC_012469.1) as a reference sequence. We did not identify and remove recombination events in the WGS alignment before constructing the phylogeny. Data on antimicrobial drug susceptibility testing performed according to British Society for Antimicrobial Chemotherapy guidelines (*11*) were available on a subset of isolates.

**Figure 1:**
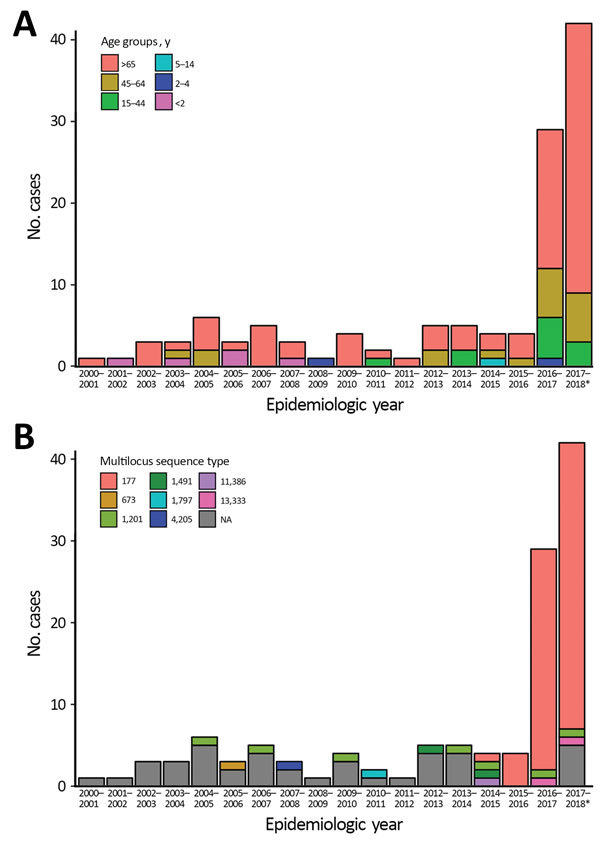
Cases of invasive pneumococcal disease (IPD) caused by serotype 7C between epidemiologic years 2000–01 and 2016–17 and an additional 42 cases reported July 1, 2017, through January 31, 2018, by (A) age group and (B) MLST sequence type, England and Wales. Surveillance is not complete for the 2017–18 epidemiologic year; figure shows only cases for the 7 months indicated. NA indicates missing sequence data.

From epidemiologic year 2000–01 through 2015–16, a total of 84,305 laboratory-confirmed IPD episodes occurred in England and Wales, including 51 serotype 7C IPD cases; an annual average of 3 cases (range 1–6) were confirmed. In 2016–17, a total of 29 cases were confirmed, compared to 5, 4, and 4 in the previous 3 years (2013–14, 2014–15, and 2015–16, respectively); an additional 42 cases were confirmed during the first 7 months of the 2017–18 epidemiologic year (Figure 1, panel A). We found no evidence of geographic or temporal clustering of cases. Cases were diagnosed across all age groups and especially in those >65 years of age (84/122, 68.9%), including 38 cases in the 65–79 age group and 46 cases in persons >80 years of age. Of the 80 cases (93.8%) diagnosed from 2000–01 through 2016–17, a total of 75 included bacteremia and 5 meningitis (in patients ages 1 month, 5 months, 6 months, 30 years, and 48 years). 

All isolates were cultured from blood or cerebrospinal fluid. Case-fatality ratio (CFR) was 25% (20/80) and increased with age: 12.5% (1/8) in children <15 years of age, 0 in the 15–44-year age group, 23% (3/13) in the 45–64-year age group, 25% (6/24) in the 65–79-year age group, and 37% (10/27) in the >80-year age group. CFR in the oldest 2 age groups was similar to the CFR for other serotypes in those age groups (23% for the 65–79-year age group and 40% for the >80-year age group).

The first serotype 7C isolate associated with ST177 appeared in 2012–13 and again in 2014–15, followed by all 4 cases in 2015–16 and 27/29 cases in 2016–17 (Figure 1, panel B). During July 2017–January 2018, a total of 35/37 sequenced 7C isolates were associated with ST177. A review of available PHE MLST data showed that ST177 was also associated with serotypes 19F (MLST derived for 25 isolates during 2002–2015) and 24F (MLST derived for 13 isolates during 2014–15).

**Figure 2:**
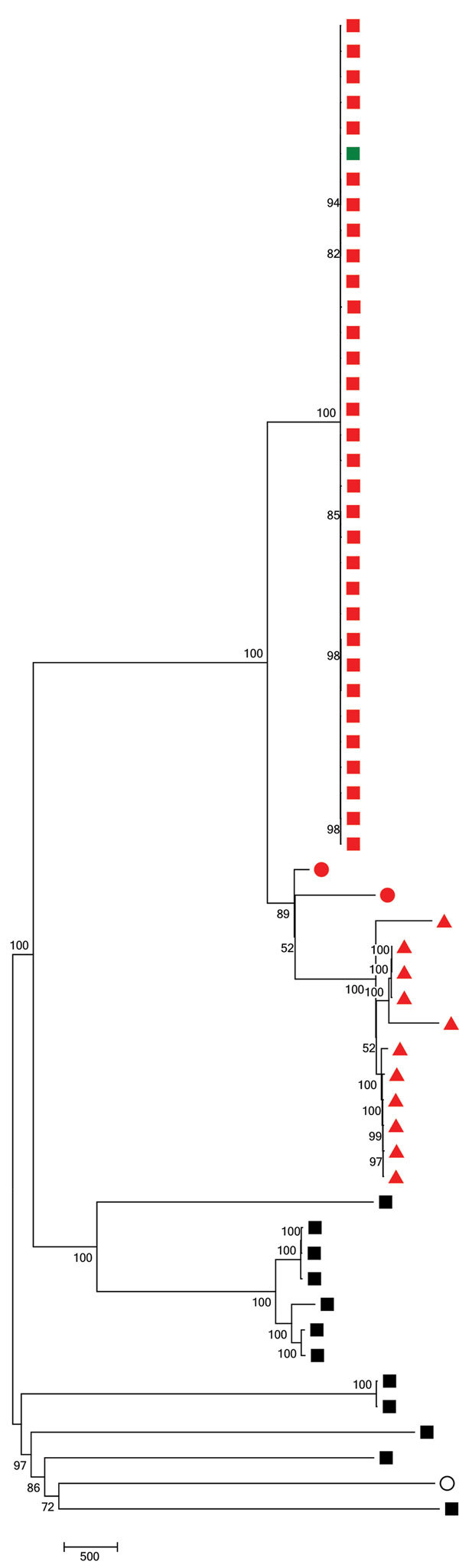
Neighbor-joining tree following single-nucleotide polymorphism analyses on 59 pneumococcal isolates collected in England and Wales during July 2005–June 2017. The percentage of replicate trees in which the associated taxa clustered together in the bootstrap test (1,000 replicates) is shown next to the branch junctions. All positions with <90% site coverage were eliminated. Missing data and ambiguous bases were allowed at any position. There were a total of 20,359 variant positions in the final dataset. Red nodes represent ST177 isolates; green node, an isolate with SLV of ST177; black nodes, non-ST177 isolates. Squares represent 7C serotype; triangles, 24F serotype; and circles 19F serotype. The open circle represents the Taiwan 19F-14 reference sequence (GenBank accession no. NC_012469.1). Scale bar shows the number of nucleotide substitutions represented by branch length. ST, sequence type.

We generated the neighbor-joining tree (Figure 2) following SNP analyses on 59 isolates, using MEGA7 using isolate Taiwan 19F-14. The tree shows that 7C-ST177 isolates had a more recent common ancestor with serotypes 19F and 24F belonging to ST177 than with non-ST177 7C isolates (except the single isolate belonging to ST11386, a single-locus variant [SLV] of ST177). The 7C-ST177 isolates were closely related, with <100 SNPs between them. We found >6,000 SNPs separating the 7C-ST177 and the non-ST177 serotype 7C (except 1 SLV of ST177 with <100 SNP distance from the ST177 isolates).

We did not detect any antimicrobial drug resistance determinants or PBP types suggesting β-lactam nonsusceptibility in the genomes of any ST177 7C isolates. MIC data were available on 13 serotype 7C isolates but only 2 were ST177; both were fully susceptible to all antimicrobial drugs tested.

## Conclusions

In England and Wales, the recent increase in IPD due to the nonvaccine serotype 7C was associated with clonal expansion of ST177. Previously, this serotype was associated with vaccine serotype 19F, although we also observed serotype 24F isolates with this ST in later years. Nearly all the increase occurred in older adults, with no evidence of change in clinical presentation or CFR when compared with the other serotypes causing IPD in the same age groups. The age-related CFRs observed in our cohort are consistent with those from other industrialized countries with established PCV programs (*12*).

Additional WGS data from historical serotypes 19F and 24F isolates may help determine whether this 7C-ST177 arose from capsular switching, which appears likely given the rapid expansion of this clone. It is also possible that the increase in serotype 24F IPD after PCV13 replaced PCV7 may be attributable to ST177 (*1*), although we did not investigate this in our study. IPD cases due to serotype 24F have increased in children (from 3 to 28 cases), adults (from 7 to 71 cases), and older adults (from 14 to 84 cases) since PCV13 implementation (*1*).

ST177 has been associated with serotype 19F, with 39/42 isolates submitted to the PubMLST isolates database associated with this serotype (https://pubmlst.org/spneumoniae/). In England and Wales, ST177 was represented exclusively by serotype 19F in elderly patients before PCV7 introduction (*13*). Because serotypes 19F and 24F are common causes of IPD, we were unable to sequence all invasive isolates. The clonal expansion of 7C-ST177 could be a consequence of natural fluctuation of nonvaccine serotypes caused by negative frequency selection (*14*). Although ST177 is the ST of the globally disseminated Portugal 19F-21 Pneumococcal Molecular Epidemiology Network clone that is multidrug resistant (*15*), we did not identify resistance determinants in any of the 7C-ST177 isolates we studied.

In summary, serotype 7C remains a rare cause of IPD in England and Wales but is linked to a sudden, rapid increase in cases of IPD because of clonal expansion of ST177, previously associated with vaccine serotype 19F. We encourage other countries to monitor pneumococcal surveillance programs for evidence of similar increases.

Technical Appendix Whole-genome sequence data used in study of invasive pneumococcal disease serotype 7C.

## References

[1] Waight PA, Andrews NJ, Ladhani SN, Sheppard CL, Slack MP, Miller E. Effect of the 13-valent pneumococcal conjugate vaccine on invasive pneumococcal disease in England and Wales 4 years after its introduction: an observational cohort study. Lancet Infect Dis. 2015;15:535–43. 10.1016/S1473-3099(15)70044-7 25801458

[2] Lund E, Henrichsen J. Laboratory diagnosis, serology and epidemiology of *Streptococcus pneumoniae.* In: Bergan T, Norris JR, editors. Methods in microbiology. 12th edition. London, New York, San Francisco: Academic Press; 1978. p. 241–62.

[3] Kapatai G, Sheppard CL, Al-Shahib A, Litt DJ, Underwood AP, Harrison TG, et al. Whole genome sequencing of Streptococcus pneumoniae: development, evaluation and verification of targets for serogroup and serotype prediction using an automated pipeline. PeerJ. 2016;4:e2477. 10.7717/peerj.2477 27672516 PMC5028725

[4] Tewolde R, Dallman T, Schaefer U, Sheppard CL, Ashton P, Pichon B, et al. MOST: a modified MLST typing tool based on short read sequencing. PeerJ. 2016;4:e2308. 10.7717/peerj.2308 27602279 PMC4991843

[5] Langmead B. Aligning short sequencing reads with Bowtie. Curr Protoc Bioinform. 2010;32: 11.7.1–11.7.14. 10.1002/0471250953.bi1107s32 PMC3010897 21154709

[6] Gupta SK, Padmanabhan BR, Diene SM, Lopez-Rojas R, Kempf M, Landraud L, et al. ARG-ANNOT, a new bioinformatic tool to discover antibiotic resistance genes in bacterial genomes. Antimicrob Agents Chemother. 2014;58:212–20. 10.1128/AAC.01310-13 24145532 PMC3910750

[7] Li Y, Metcalf BJ, Chochua S, Li Z, Gertz RE Jr, Walker H, et al. Penicillin-binding protein transpeptidase signatures for tracking and predicting β-lactam resistance levels in *Streptococcus pneumoniae.* MBio. 2016;7:e00756–16. 10.1128/mBio.00756-16 27302760 PMC4916381

[8] Kumar S, Stecher G, Tamura K. MEGA7: Molecular Evolutionary Genetics Analysis version 7.0 for bigger datasets. Mol Biol Evol. 2016;33:1870–4. 10.1093/molbev/msw054 27004904 PMC8210823

[9] Saitou N, Nei M. The neighbor-joining method: a new method for reconstructing phylogenetic trees. Mol Biol Evol. 1987;4:406–25. 3447015 10.1093/oxfordjournals.molbev.a040454

[10] Kapatai G, Sheppard CL, Troxler LJ, Litt DJ, Furrer J, Hilty M, et al. Pneumococcal 23B molecular subtype identified using whole genome sequencing. Genome Biol Evol. 2017;9:2122–35. 10.1093/gbe/evx092 28910966 PMC5581491

[11] A guide to sensitivity testing. Report of the Working Party on Antibiotic Sensitivity Testing of the British Society for Antimicrobial Chemotherapy. J Antimicrob Chemother. 1991;27 Suppl D:1–50. 1874686

[12] Marrie TJ, Tyrrell GJ, Majumdar SR, Eurich DT. Effect of age on the manifestations and outcomes of invasive pneumococcal disease in adults. Am J Med. 2018;131:100.e1–7. 10.1016/j.amjmed.2017.06.039 28803139

[13] Pichon B, Bennett HV, Efstratiou A, Slack MP, George RC. Genetic characteristics of pneumococcal disease in elderly patients before introducing the pneumococcal conjugate vaccine. Epidemiol Infect. 2009;137:1049–56. 10.1017/S0950268808001787 19161642

[14] Corander J, Fraser C, Gutmann MU, Arnold B, Hanage WP, Bentley SD, et al. Frequency-dependent selection in vaccine-associated pneumococcal population dynamics. Nat Ecol Evol. 2017;1:1950–60. 10.1038/s41559-017-0337-x 29038424 PMC5708525

[15] Sá-Leão R, Tomasz A, Sanches IS, Brito-Avô A, Vilhelmsson SE, Kristinsson KG, et al. Carriage of internationally spread clones of *Streptococcus pneumoniae* with unusual drug resistance patterns in children attending day care centers in Lisbon, Portugal. J Infect Dis. 2000;182:1153–60. 10.1086/315813 10979912

