# Supplementary material for: Rapid Spread of Pneumococcal Nonvaccine Serotype 7C Previously Associated with Vaccine Serotype 19F, England and Wales
Source: Emerg Infect Dis. 2018 Oct;24(10):1919–22. doi: 10.3201/eid2410.180114 (PMC6154145; doi:10.3201/eid2410.180114)
Supplement: Technical Appendix — Whole-genome sequence data used in study of invasive pneumococcal disease serotype 7C. [file 18-0114-Techapp-s1.pdf]

Article DOI: https://doi.org/10.3201/eid2410.180114

Rapid Spread of Pneumococcal Nonvaccine Serotype 7C Previously Associated with Vaccine Serotype 19F, England and Wales

Technical Appendix Table. Isolate designations and European Nucleotide Archive (ENA) accession numbers for whole-genome sequence data used in study of invasive pneumococcal disease serotype 7C

Sample no.	ENA accession no.	Serotype	Sequence type
PHESP000001	ERS2029289	24F	177
PHESPD0753	ERS1194051	24F	177
PHESPD0753	ERS1194051	24F	177
PHESPV0348	ERS1194516	24F	177
PHESPV0718	ERS1194886	24F	177
PHESPV0725	ERS1194893	24F	177
PHESPV0803	ERS1194971	24F	177
PHESPV1151	ERS1195319	24F	177
PHESPV1385	ERS1195551	24F	177
PHESPV1386	ERS1195552	24F	177
PHESPV1528	ERS1195692	24F	177
PHESPV1737	ERS1195901	24F	177
PHESPV1747	ERS1195911	24F	177
PHESPD0806	ERS1194104	19F	177
PHESPV1262	ERS1195430	19F	177
PHESP000002	ERS2029290	7C	177
PHESP000003	ERS2029291	7C	177
PHESP000004	ERS2029292	7C	177
PHESP000005	ERS2029293	7C	177
PHESP000006	ERS2029294	7C	13333
PHESP000007	ERS2029295	7C	177
PHESP000008	ERS2029296	7C	177
PHESP000009	ERS2029297	7C	177
PHESP000010	ERS2029298	7C	177
PHESP000011	ERS2029299	7C	1201
PHESP000012	ERS2029300	7C	177
PHESP000013	ERS2029301	7C	177
PHESP000014	ERS2029302	7C	177
PHESP000015	ERS2029303	7C	177
PHESP000016	ERS2029304	7C	177
PHESP000017	ERS2029305	7C	177
PHESP000018	ERS2029306	7C	177
PHESP000019	ERS2029307	7C	177
PHESP000020	ERS2029308	7C	177
PHESP000021	ERS2029309	7C	177
PHESP000022	ERS2029310	7C	177
PHESP000023	ERS2029311	7C	177
PHESP000024	ERS2029312	7C	177
PHESP000025	ERS2029313	7C	177
PHESP000026	ERS2029314	7C	177
PHESP000027	ERS2029315	7C	177
PHESP000028	ERS2029316	7C	177
PHESP000029	ERS2029317	7C	177
PHESP000030	ERS2029318	7C	177
PHESP000031	ERS2029319	7C	177
PHESP000032	ERS2029320	7C	177
PHESP000033	ERS2029321	7C	177
PHESPD0208	ERS1193506	7C	4205
PHESPD0276	ERS1193574	7C	1201
PHESPD0320	ERS1193618	7C	1797

Sample no.	ENA accession no.	Serotype	Sequence type
PHESPD0350	ERS1193648	7C	1491
PHESPV0498	ERS1194666	7C	1201
PHESPV0975	ERS1195143	7C	1491
PHESPV0993	ERS1195161	7C	11386
PHESPV1016	ERS1195184	7C	1201
PHESPV1052	ERS1195220	7C	177
PHESPV1594	ERS1195758	7C	177
PHESPV1945	ERS1196109	7C	1201
PHESPV2010	ERS1196174	7C	1201
PHESPV2043	ERS1196207	7C	673
